# Energy-Stress-Mediated AMPK Activation Promotes GPX4-Dependent Ferroptosis through the JAK2/STAT3/P53 Axis in Renal Cancer

**DOI:** 10.1155/2022/2353115

**Published:** 2022-10-04

**Authors:** Yanze Li, Ye Zhang, Qiangmin Qiu, Lei Wang, Hu Mao, Juncheng Hu, Zhiyuan Chen, Yang Du, Xiuheng Liu

**Affiliations:** Department of Urology, Renmin Hospital of Wuhan University, Wuhan, 430060 Hubei, China

## Abstract

Energy stress is an unfavorable condition that tumor cells are often exposed to. Ferroptosis is considered an emerging target for tumor therapy. However, the role of ferroptosis in energy stress in renal cancer is currently unknown. In this study, we found that glucose deprivation significantly enhanced GPX4-dependent ferroptosis through AMPK activation. Further, AMPK activation suppressed GPX4 expression at the transcriptional level through the upregulation of P53 expression. Additionally, the inactivation of JAK2/STAT3 transcriptionally promoted P53 expression, thereby promoting AMPK-mediated GPX4-dependent ferroptosis. In conclusion, energy stress promotes AMPK-mediated GPX4-dependent erastin-induced ferroptosis in renal cancer through the JAK2/STAT3/P53 signaling axis.

## 1. Introduction

Cancer cells often exhibit alternative pathways for energy metabolism for growth and proliferation. Findings from extensive reports have revealed that adaptive changes in energy metabolism play vital roles in cancer survival [1]. The upregulation of glycolysis is widely considered a major catabolic process alteration in cancer cells in response to energy stress, referred to as the Warburg effect [2]. Because of the Warburg effect, glucose acts as a primary metabolic fuel for several tumors [3]. Rapid growth and a relatively limited energy supply often expose tumors to energy stress. AMP-activated protein kinase (AMPK) is a critical energy sensor that can sense the cellular energy status [4]. Energy stress increases the AMP/ATP ratio in cells. In response, AMPK is activated to restore the energy balance, primarily through the inhibition of lipid and protein synthesis as well as the promotion of glucose uptake and glycolysis [4]. Thus, cancer cells can adapt to these harsh environmental conditions.

Ferroptosis is a novel form of regulated cell death. Unlike apoptosis, autophagy, or necrosis, ferroptosis is characterized by lethal iron-dependent lipid peroxidation accumulation. Emerging evidence has shown that ferroptosis is related to various human diseases and pathological conditions, such as degenerative diseases, stroke, ischemia-reperfusion injury, and cancer [5]. The sensitivity to ferroptosis is associated with many factors, and metabolic aberration is a major part of the same [6, 7]. Therefore, regulation of the expression of genes or kinases involved in these metabolic pathways can partially control ferroptosis sensitivity. System Xc- and glutathione peroxidase 4 (GPX4) are widely known to be required for ferroptosis prevention. For example, the inhibition of system Xc- can block cystine/glutamate transport, leading to cellular glutathione depletion. Glutathione is a major antioxidant and can be utilized by GPX4 to reduce lipid peroxidation. Thus, the depletion of glutathione eventually induces ferroptosis [8]. However, the relationship between ferroptosis and energy metabolism remains elusive. In particular, this has led to some controversies and discussions in previous reports [9, 10].

In recent years, Janus kinase (JAK)/signal transducer and activator of transcription (STAT) pathway has been discovered as an intracellular signal transduction pathway closely related to cytokines [11]. It was reported that JAK/STAT3 signaling pathway was involved in many biological processes such as cell proliferation, differentiation, apoptosis, and immune regulation [11]. JAK2/STAT3, an important isoform of JAK/STAT, is associated with proliferation, migration, metastasis, and cachexia in many cancers [12, 13]. Yet, the role of JAK2/STAT3 signaling pathway in energy stress condition is still unknown.

Renal cancer represents the sixth-most common diagnosed cancer in men and the eighth-most common cancer in women, accounting for 5% and 3% of all new cancer cases in 2020, respectively [14]. However, despite the application of various treatments, such as targeted therapy and immune therapy, the unfavorable outcomes in renal cancer suggest the urgency of the need for novel therapeutics [15]. Renal cancer cells are relatively susceptible to ferroptosis, suggesting a great potential for antitumor therapy [16]. Considering that the underlying mechanisms of energy stress-mediated ferroptosis in cancer remain ambiguous, we explore the effects of energy stress on ferroptosis in renal cancer.

## 2. Methods and Materials

### 2.1. Cell Culture

Renal cell cancer cell lines 786-O and A498 were purchased from ATCC (American Type Culture Collection, Manassas, VA, USA). The cells were cultured in RPMI 1640 medium supplemented with 10% fetal bovine serum (FBS, GIBCO, MA, USA) under 5% CO_2_ at 37°C. For glucose starvation treatment, cells were cultured in normal medium for 24 h and washed twice with PBS, and the medium was replaced with a glucose-free medium supplemented with 10% FBS.

Cells were treated with the ferroptosis inducer erastin (1.5 *μ*M) (MedChemExpress, China) for 24 h, the ferroptosis inhibitor ferrostatin-1 (2 *μ*M) (Fer-1, MedChemExpress) for 24 h, the AMPK inhibitor Compound C (COM C) (10 *μ*M) (MedChemExpress) for 24 h, the AMPK activator AICAR (2 mM) (MedChemExpress) for 24 h, Pifithrin-*α* (5 *μ*M) (PFT-*α*, MedChemExpress) for 24 h, the apoptosis inhibitor Z-VAD-FMK (20 *μ*M) (MedChemExpress), and the necroptosis inhibitor necrostatin-1 (2 *μ*M) (Nec-1, MedChemExpress).

### 2.2. siRNA Construction and Cell Transfection

siRNAs were constructed and purchased from Wuhan Viraltherapy Technologies Co. Ltd. Cells were transfected with the siRNAs using Lipofectamine 2000 (Invitrogen, China) for at least 48 h. If not specifically stated, siRNA negative control was transfected to the other groups as control.

### 2.3. Plasmid Construction and Cell Transfection

Plasmids were extracted using the Small Plasmid Extraction Kit (EM101, TransGen Biotech, China), and single colonies were then amplified. The bacterial culture fluid was subjected to sequencing. 293 T cells were cotransfected with pLVX-GPX4-ZsGreen-Puro and pLVX-JAK2-ZsGreen-Puro (recombinant plasmids) using the Lentivirus Packaging Kit (R003, Wuhan Viraltherapy Technologies Co., Ltd.) to obtain high-titer lentiviruses containing the target genes (rLV-GPX4, rLV-JAK2). Lentiviruses loaded with the recombinant plasmids were transfected into cells at an MOI of 20 for 48 h. Lentivirus-infected cells were selected using DMEM containing 10 *μ*g/mL puromycin.

### 2.4. Cell Death Assay

Cell death was measured by propidium iodide (PI) staining using a flow cytometer. After subjecting to different treatments, the cells, including floating cells, were collected and stained with 5 *μ*g/mL PI, following which the percentage of positive cells was counted and analyzed on a flow cytometer (BD Accuri C6).

### 2.5. Lipid Peroxidation Assay

Malondialdehyde (MDA) is one of the end products of lipid peroxidation. The lipid peroxidation levels were evaluated using the MDA assay kit (Nanjing Jiancheng Bioengineering Institute, China). An assay was conducted according to the manufacturer's instructions. The absorbance was measured at 525 nm using a microplate reader (Bio-Rad Laboratories, Redmond, WA, USA) [17]. The MDA levels were presented as a ratio of the absorbance value of the control group.

### 2.6. Reduced Glutathione (GSH) Level Measurement

GSH levels were assayed using the GSH Assay Kit (Nanjing Jiancheng Bioengineering Institute, China) according to the manufacturer's instructions. Absorbance was measured at 405 nm using a microplate reader (Bio-Rad). The GSH levels were presented as a ratio to the absorbance value of the control group.

### 2.7. Cellular Iron Level Measurement

Cellular iron levels were detected by the Iron Assay kit (Nanjing Jiancheng Bioengineering Institute, China) according to the manufacturer's instructions. Absorbance was measured at 520 nm using a microplate reader (Bio-Rad). The cellular iron levels were presented as a ratio of the absorbance value of the control group.

### 2.8. Western Blotting Analysis

Total proteins were collected by lysing the cells with the RIPA Lysis Buffer (Beyotime, China) and quantified using the BCA protein assay kit (Beyotime). The proteins were mixed with a loading buffer and denatured by heating in boiling water. The protein samples were separated on 10% SDS-PAGE gels and transferred to PVDF membranes. After blocking with 5% nonfat milk and washing with TBST buffer, the membranes were treated overnight with primary antibodies at 4°C. The primary antibodies used in our study were as follows: rabbit polyclonal anti-AMPK alpha antibody (AMPK *α*, 1: 1000, Affinity), rabbit polyclonal anti-phospho-AMPK alpha (Thr172) antibody (p-AMPK *α*, 1 : 1000, Affinity), rabbit polyclonal anti-GPX4 antibody (1 : 1000, Affinity), rabbit polyclonal anti-P53 antibody (1 : 1000, Affinity), rabbit polyclonal anti-STAT3 antibody (1: 1000, Affinity), rabbit polyclonal anti-phospho-STAT3 (Tyr705) antibody (p-STAT3, 1: 1000, Affinity), rabbit polyclonal anti-JAK2 antibody (1: 1000, Affinity), rabbit polyclonal anti-phospho-JAK2 (Tyr931) antibody (p-JAK2, 1: 1000, Affinity), and rabbit polyclonal anti-GAPDH antibody (1: 4000, Affinity). After washing with TBST, the membranes were then treated with a secondary antibody (HRP-conjugated antirabbit secondary antibodies, 1 : 5000, Boster Biological Technology Co. Ltd) at room temperature for 2 h. Finally, the membranes were evaluated using a chemiluminescence system (Bio-Rad).

### 2.9. Quantitative Real-Time PCR

Total RNA was extracted by TRIzol, and the RNA concentration was measured using a spectrophotometer. cDNA was synthesized using the PrimeScript™ RT Reagent Kit (Takara) according to the manufacturer's instructions. qRT-PCR was conducted using a Bio-Rad real-time PCR system with the SYBR Green PCR Master Mix (Takara). The expression levels are presented as a ratio relative to the expression of GAPDH. Data analysis was performed using the 2^−*ΔΔ*Ct^ method. The primers used in the study are shown below.

*GPX4* (Forward) 5′-GAGGCAAGACCGAAGTAAACTAC-3′

(Reverse) 5′- CCGAACTGGTTACACGGGAA-3′

*TP53* (Forward) 5′-GAGATGTTCCGAGAGCTGA-3′

(Reverse) 5′-TCAGCTCTCGGAACATCTC-3′

*GAPDH* (Forward) 5′-AACGGATTTGGTCGTATTGGG-3′

(Reverse) 5′-CCTGGAAGATGGTGATGGGAT-3′

### 2.10. Transmission Electron Microscope (TEM)

Cells were collected using trypsin. After centrifugation, the cells were fixed in 2.5% glutaraldehyde at 4°C for 24 h. Then, the cells were fixed in 2% osmium tetroxide at room temperature. After dehydration in increasing concentrations of ethanol, the samples were embedded in epoxy resin and cut into 60-nm ultrathin sections. Eventually, the sections were stained with 1% uranyl acetate and observed using a Hitachi TEM system (HT7800).

### 2.11. Bioinformatics Analysis

The mRNA expression profiles and corresponding clinical data of 537 patients with renal cancer were retrieved from TCGA (TCGA-KIRC, up to October 29, 2021), and data for 258 ferroptosis-related genes were retrieved from the FerrDb database. Differentially expressed genes (DEGs) between the AMPK high-expression and low-expression groups were screened using the “limma” R package with a false discovery rate<0.05, and following this, the intersecting genes between DEGs and ferroptosis-related genes were identified. Among the intersecting genes, the ones for which the expression had a correlation >0.8 with the AMPK score were selected for visualization with a protein-protein interaction (PPI) network using STRING 11.5. Coexpression analysis was performed on ENCORI [18]. The “survival” and “survminer” R packages were used to perform univariate and multivariate COX analyses, and Kaplan-Meier (K-M) curves for overall survival were plotted using the “ggsurvplot2” R package. The variance in clinicopathological characteristics between subgroups was presented using the “ggpubr” R package.

### 2.12. Tissue Collection

Sixty-one pairs of renal cancer tissues and adjacent normal tissues were collected from patients undergoing ectomy of renal cancer carcinoma in Renmin Hospital of Wuhan University from January 2021 to December 2021. All research protocols were approved by the Ethics Committee of Renmin Hospital of Wuhan University.

### 2.13. Immunohistochemical Assay

Paraffinized blocks of tissues were cut into sections. The sections were deparaffinized and hydrated and then treated with a primary antibody and secondary antibody sequentially. Finally, the sections were stained with 3,3′-diaminobenzidine, a chromogenic agent (Dako Corp, Carpinteria, CA, USA). The primary antibodies used here were the same as those mentioned in the previous section, with a dilution of 1 : 50. The sections were scored independently by two experienced pathologists in a single-blind manner. The method of semiquantitative scoring has been described in a previous study [19]. A score ≤4 was considered to represent low expression, and a score>4 was considered to represent high expression.

### 2.14. Statistical Analysis

All data are presented as mean ± SD. Statistical analysis involved one-way ANOVA, chi-square test, and Student's *t*-test performed using GraphPad Prism 7. Unless specified otherwise, *p*˂0.05 was considered to represent a statistically significant difference.

## 3. Results

### 3.1. Energy Stress Promotes Ferroptotic Cell Death in Renal Cancer Cells

**Figure 1:**
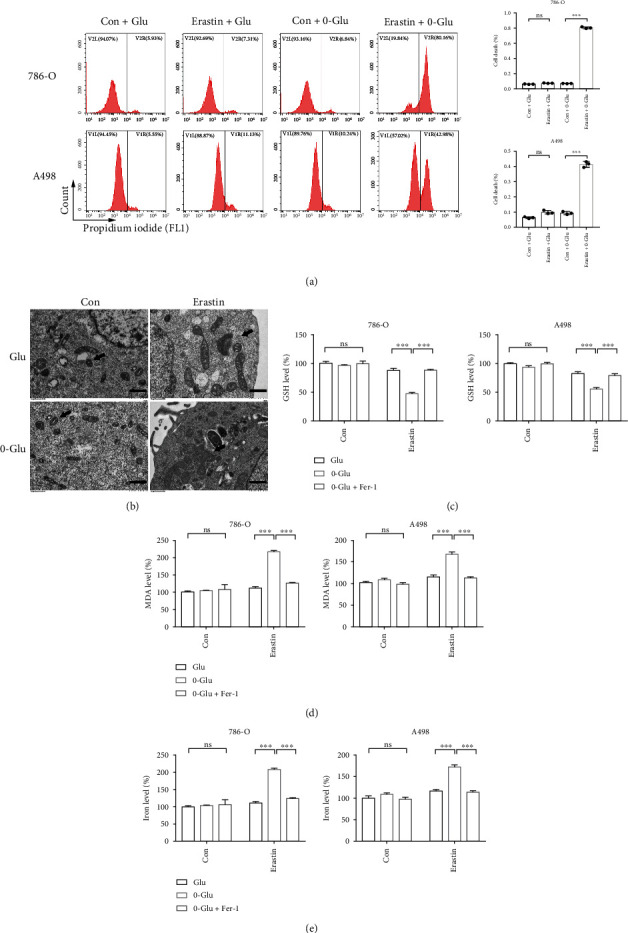
Energy stress promotes ferroptotic cell death in renal cancer cells. (a) Cell death measurement shows 0-Glu promotes erastin-induced cell death. (b) TEM was used to observe morphological changes of cellular ultrastructure. Cells in 0-Glu and erastin treatment group present aberrant mitochondria. Black arrows indicate mitochondria. Scale bars: 1 *μ*m. Independent experiments were repeated three times and representative data were shown. (c–e) 0-Glu and erastin treatment-induced GSH deletion, MDA generation, and cellular iron elevation are rescued by Fer-1. Glu: glucose concentration of RPMI 1640 medium. 0-Glu: 0 mM glucose. Data shown represent mean ± SD from at least three independent experiments. Comparisons were performed using Student's *t*-test. Fer-1: ferrostatin-1; ns: not significant. ^∗∗∗^*p* < 0.001.

Glucose is the main source of energy for cancer cells. We simulated energy stress by glucose deprivation. Glucose deprivation significantly aggravated erastin-induced cell death (Figure 1(a)). To determine whether the increased cell death was ferroptotic cell death, we observed the cells via TEM and found that in response to treatment with erastin, the cells developed typical morphological features of ferroptosis, such as shrinkage of the mitochondria, increase in membrane density, and disappearance of mitochondrial cristae. Notably, these changes were more drastic after glucose deprivation (Figure 1(b)). GSH depletion and increase in lipid peroxidation were the key biochemical changes in ferroptosis. We demonstrated that glucose deprivation significantly decreased the intracellular GSH level while significantly increasing the MDA level and cellular iron level, and this trend could be reversed by Fer-1, a potent inhibitor of ferroptosis (Figures 1(c)–1(e)). The above results suggest that energy stress promotes ferroptosis in renal cancer cells.

### 3.2. Energy Stress Promotes Ferroptotic Cell Death through AMPK Activation

**Figure 2:**
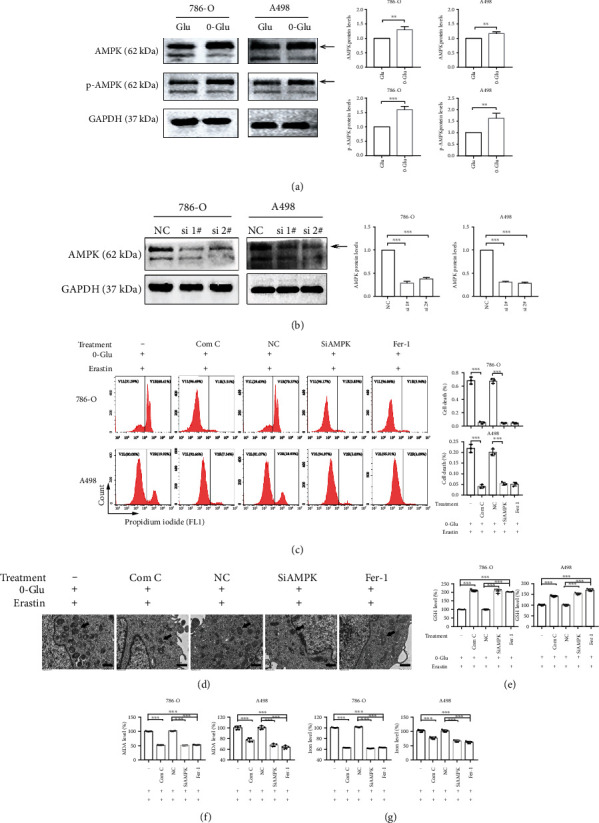
Energy stress promotes ferroptotic cell death through AMPK activation. (a and b) Western blotting assay verifies the activation of AMPK after 0-Glu treatment and the knockdown efficacy of two siAMPK. (c) Cell death measurement shows the alleviation of cell death by Com C, siAMPK, and Fer-1 after 0-Glu and erastin treatment. (d) TEM images shows that Com C, siAMPK, and Fer-1 treatments recover mitochondrial morphology. Black arrows indicate mitochondria. Scale bars: 1 *μ*m. Independent experiments were repeated three times, and representative data were shown. (e–g) Com C, siAMPK, and Fer-1 treatments reverse the changes of GSH, MDA, and cellular iron level caused by 0-Glu and erastin treatment. Data shown represent mean ± SD from at least three independent experiments. Comparisons were performed using Student's *t*-test and one-way ANOVA. NC: negative control; Com C: Compound C; Fer-1: ferrostatin-1. ^∗∗^*p* < 0.01 and ^∗∗∗^*p* < 0.001.

AMPK, as an energy receptor, is known to be activated in response to energy stress. We next explored whether the activation of AMPK is involved in the promotion of ferroptosis by energy stress. The western blotting results confirmed that the activation of AMPK phosphorylation (p-AMPK) increased after glucose deprivation (Figure 2(a)). Next, we constructed two siAMPKs, of which 1#siAMPK was more effective and used in subsequent studies (Figure 2(b), Supplementary Figure 2). COM C is a potent selective AMPK inhibitor. After glucose deprivation, the inhibition of AMPK by COM C or siAMPK remarkably rescued erastin-induced ferroptosis, as observed with Fer-1 (Figure 2(c)). The TEM results also showed that the changes in mitochondrial size, membrane density, and mitochondrial cristae caused by glucose deprivation and erastin treatment were alleviated after the inhibition or knockdown of AMPK, and these changes were also identical to those observed after treatment with Fer-1 (Figure 2(d)). Similarly, GSH, MDA, and cellular iron levels reverted after AMPK inhibition or knockdown (Figures 2(e)–2(g)). Therefore, the inhibition or knockdown of AMPK reversed the erastin-induced ferroptosis enhanced by glucose deprivation. Collectively, energy stress promotes ferroptotic cell death through AMPK activation in renal cancer cells.

### 3.3. GPX4 Overexpression Alleviates Energy-Stress-Mediated Ferroptotic Cell Death

**Figure 3:**
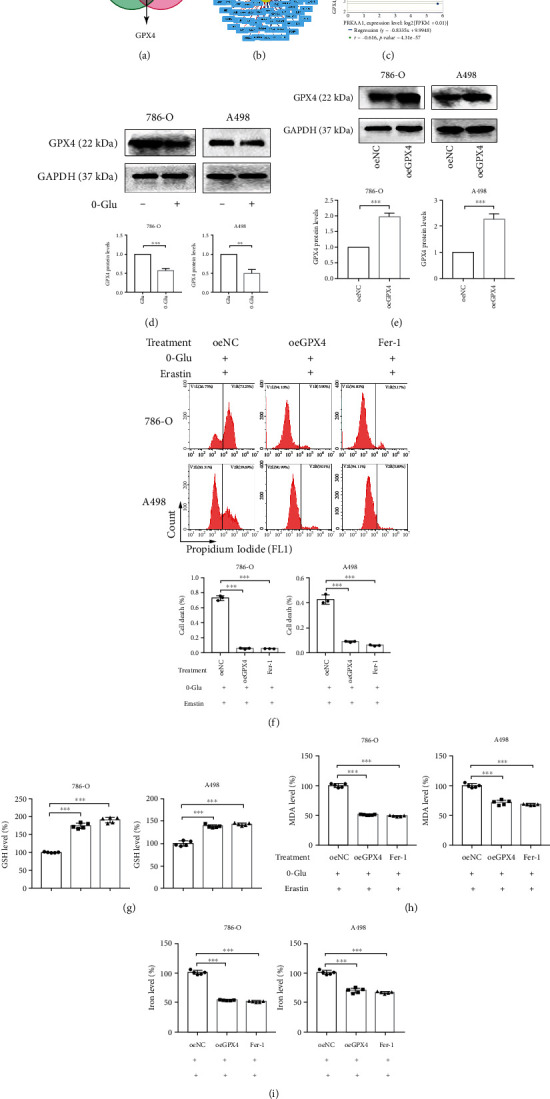
GPX4 overexpression alleviates energy-stress-mediated ferroptotic cell death. (a) VENN diagram shows *GPX4* as a ferroptosis-related differentially expressed genes. (b and c) PPI network and coexpression analysis shows the correlation between AMPK and GPX4. (d) Western blotting assay presents a decrease of GPX4 expression after glucose-starvation. (e) Western blotting assay verifies the effect of GPX4 overexpression. (f) Cell death measurement in cells treated with indicated treatments. Independent experiments were repeated three times, and representative data were shown. (g–i) The measurement of GSH, MDA, and cellular iron in cells treated with oeGPX4 or Fer-1 after o-Glu and erastin treatment. Data shown represent mean ± SD from at least three independent experiments. Comparisons were performed using Student's *t*-test and one-way ANOVA. oeNC: overexpression negative control; Fer-1: ferrostatin-1. ^∗∗^*p* < 0.01 and ^∗∗∗^*p* < 0.001.

Next, we used bioinformatics analysis for identifying 102 differentially expressed ferroptosis-related genes between the AMPK (encoded by *PRKAA*) high- and low-expression groups using data from TCGA. Surprisingly, we found that GPX4, one of the most critical enzymes for ferroptosis, featured among these 102 genes (Figure 3(a)). Moreover, by PPI and coexpression analyses, we found that GPX4 expression showed high negative correlation with AMPK expression (Figures 3(b) and 3(c)).

Therefore, we further confirmed the role of GPX4 in energy-stress-mediated ferroptosis. Western blotting results showed a decrease in the GPX4 protein level after glucose deprivation (Figure 3(d)). Following this, we constructed GPX4 overexpression (oeGPX4) renal cancer cell lines (Figure 3(e)). Ferroptosis induced by glucose deprivation and erastin treatment decreased significantly after GPX4 overexpression, as observed after Fer-1 treatment as well (Figure 3(f)). In parallel, the GSH, MDA, and cellular iron levels were ameliorated by GPX4 overexpression (Figures 3(g)–3(i)). Hence, the results of bioinformatics analysis predicted that GPX4 may be involved in energy-stress-mediated ferroptosis, and findings from the rescue assays further confirmed that GPX4 overexpression alleviated energy-stress-mediated ferroptotic cell death.

### 3.4. AMPK Activation Enhances GPX4-Dependent Ferroptosis

**Figure 4:**
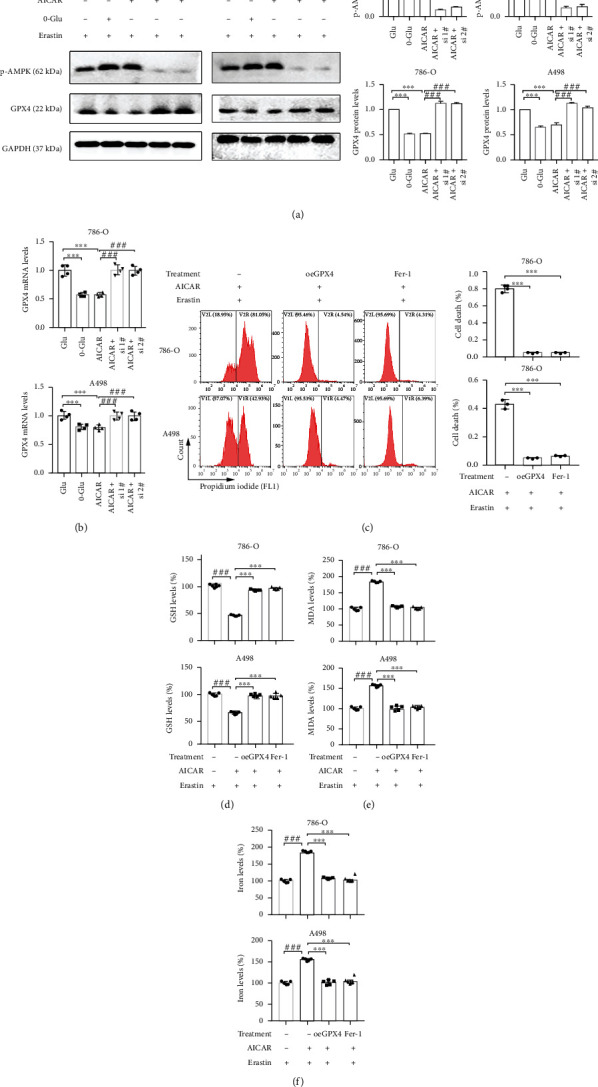
AMPK activation enhances GPX4-dependent ferroptosis. (a and b) Western blotting assay and qRT-PCR show the changes of p-AMPK and GPX4 protein levels and *GPX4* mRNA level in cells treated with AICAR and siAMPK. All groups were treated with erastin. (c) Cell death measurement presents the effect of AICAR and oeGPX4 on erastin-induced cell death. Independent experiments were repeated three times and representative data were shown. (d–f) The measurement of GSH, MDA, and cellular iron levels in cells treated with AICAR and oeGPX4. Data shown represent mean ± SD from at least three independent experiments. Comparisons were performed using Student's *t*-test and one-way ANOVA. Fer-1: ferrostatin-1. ^∗∗^*p* < 0.01, ^∗∗∗^*p* < 0.001, ^##^*p* < 0.01, and ^###^*p* < 0.001.

The results indicate that the activation of AMPK may regulate ferroptosis through the regulation of GPX4. Next, we explored the relationship between AMPK activation and GPX4-dependent ferroptosis via the pharmacological activation of AMPK. AICAR, an adenosine analog and AMPK activator, was used. The phosphorylation level of AMPK after treatment with AICAR was confirmed to be the same as that after glucose deprivation treatment, and likewise, the protein level of GPX4 declined significantly upon AICAR treatment. Nevertheless, this decline was reversed after AMPK knockdown (Figure 4(a)). To exclude off target effect of siRNA, two siAMPK was used. The same trend was also observed at the mRNA level (Figure 4(b)), suggesting that the phosphorylation-mediated activation of AMPK could reduce GPX4 expression at the transcriptional level. In terms of ferroptosis-related phenotypes, AICAR similarly increased erastin-induced ferroptosis, whereas cell survival was recovered in response to GPX4 overexpression (Figure 4(c)). Also, we demonstrated that the intracellular GSH levels had reduced significantly, and MDA and cellular iron levels had increased upon AICAR treatment, whereas these trends had reversed after GPX4 overexpression (Figures 4(d)–4(f)). The above results suggest that AMPK activation enhances GPX4-dependent ferroptosis.

### 3.5. AMPK High + GPX4 Low Expression Signature Is a Predictor of a Favorable Prognosis of Renal Cancer

**Figure 5:**
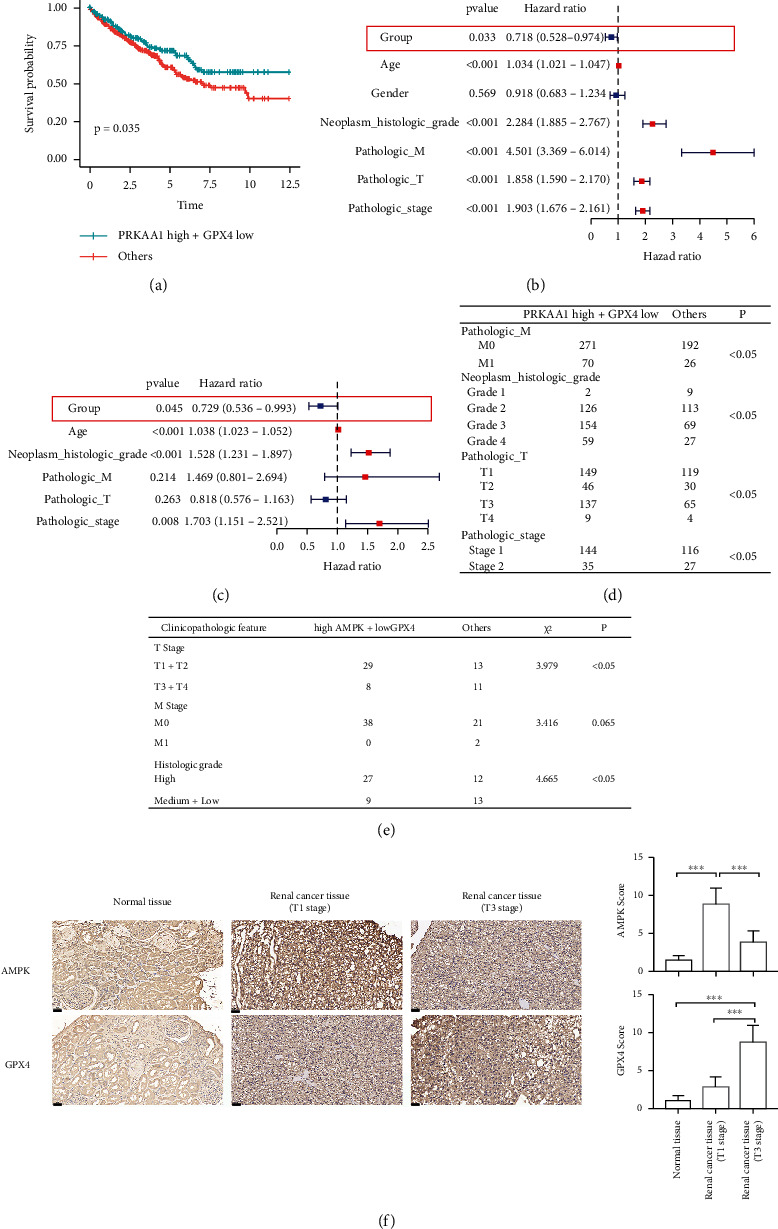
AMPK high+GPX4 low expression signature is a predictor of favorable prognosis of renal cancer. (a) K-M curves for the overall survival of patients in the AMPK high+GPX4 low expression group and others. (b and c) Forest map of univariate and multivariate COX regression analysis regarding overall survival. (d) The table shows the difference of clinicopathologic features between different group based on TCGA-KIRC dataset. (e) The table shows the information of 61 specimens we collected. Data shown represent mean ± SD from at least three independent experiments. Comparisons were performed using chi-square test. (f) Representative images present AMPK and GPX4 expression in tissues of different stage of renal cancer (20×, scale bars: 50 *μ*m). Independent experiments were repeated three times, and representative data were shown.

Considering the previous results, we categorized patients into a high AMPK (encoded by *PRKAA*) + low GPX4 expression group and others group. K-M curve analysis showed that patients with high AMPK and low GPX4 expression had better overall survival (Figure 5(a)). Findings from the univariate and multivariate COX analyses suggested that high AMPK + low GPX4 expression was a low-risk prognostic factor (Figures 5(b) and 5(c)). In addition, patients with high AMPK and low GPX4 expression typically had lower pathological T and M grades as well as neoplasm histological grade and pathological stage (Figure 5(d), Supplementary Table 1). After statistical analysis of the data obtained from our specimens, we achieved a consistent result (Figure 5(e) and 5(f)). We showed representative images in adjacent normal tissue (Figure 5(f) left) and renal cancer tissue of T1 stage (Figure 5(f) middle) and T3 stage (Figure 5(f) right).

### 3.6. AMPK Promotes GPX4-Dependent Ferroptosis Partly through the Upregulation of P53

**Figure 6:**
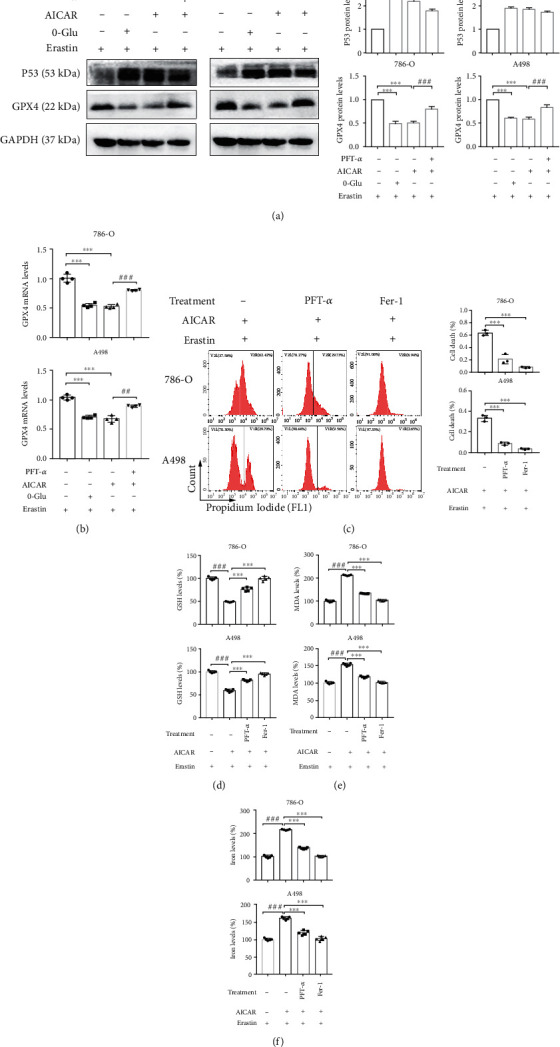
AMPK promotes GPX4-dependent ferroptosis partly through upregulation of p53. (a) Western blotting assay shows the expression of P53 and GPX4 after indicated treatments. (b) qRT-PCR presents the mRNA levels of *GPX4* after indicated treatments. (c–f) The measurements of cell death, GSH, MDA, and cellular iron in cells with indicated treatments. Independent experiments were repeated three times, and representative data were shown. Data shown represent mean ± SD from at least three independent experiments. Comparisons were performed using Student's *t*-test and one-way ANOVA. Fer-1: ferrostatin-1; ns, not significant. ^∗∗∗^*p* < 0.001 and ^###^*p* < 0.001.

*TP53* is widely known as a critical oncogene. Recently, an increasing number of studies have focused on the role of P53 in ferroptosis [20]. In this study, we investigated whether P53 was involved in AMPK-mediated GPX4-dependent ferroptosis. We observed that after glucose deprivation and AICAR treatment, the P53 levels were significantly elevated, whereas the GPX4 levels were significantly lowered. In contrast, upon treatment with PFT-*α* (an inhibitor of P53), the protein levels of GPX4 were restored (Figure 6(a)). Similarly, the mRNA levels of GPX4 also showed the same trend (Figure 6(b)). Considering the previous results, AMPK activation was suggested to transcriptionally inhibit GPX4 expression, partly through P53 upregulation. Next, we focused on the role of P53 in ferroptosis. It was demonstrated that after treatment with PFT-*α*, the increase in ferroptosis caused by erastin and AICAR was alleviated, intracellular GSH levels were elevated, MDA levels were lowered, and cellular iron levels were also reduced (Figures 6(c)–6(f)). Considering that AMPK and P53 are involved in the regulation of apoptosis in some cancers, we also performed experiments to exclude the potential of apoptosis. Firstly, the level of apoptosis hallmark cleaved caspase-3 was detected, and the results demonstrated that energy stress did not induce apoptosis in the context (Supplementary Figure 1A). Next, the cell death assay indicated that apoptosis inhibitor and necroptosis could not alleviate the cell death caused by AMPK activation and erastin (Supplementary Figure 1B). Moreover, the GSH, MDA, and cellular iron levels could not all be reversed by the inhibition of apoptosis and necroptosis either (Supplementary Figure 1C-1E). These results confirmed that the upregulation of P53 contributes to AMPK-mediated GPX4-dependent ferroptosis.

### 3.7. JAK2/STAT3 Signaling Pathway Inactivation Upregulates the Expression of P53, which Participates in AMPK-Mediated GPX4-Dependent Ferroptosis

**Figure 7:**
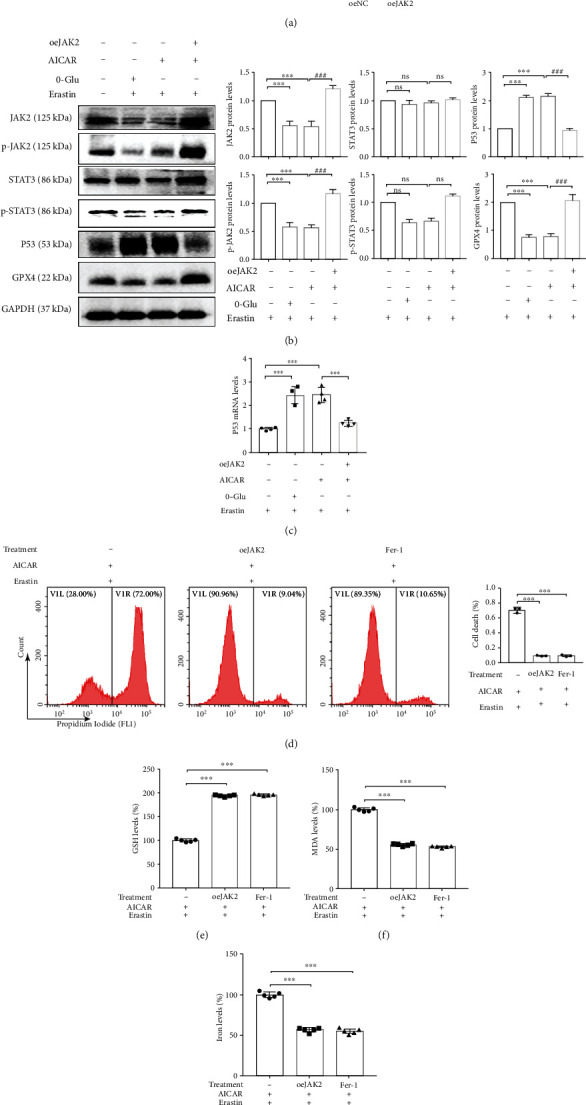
JAK2/STAT3 signaling pathway inactivation upregulates P53 expression participating AMPK-mediated GPX4-dependent ferroptosis. 786-O cells were transfected with a JAK2 overexpression plasmid. (a and b) Western blotting assay shows the effect of JAK2 overexpression and the expression of JAK2, p-JAK2, STAT3, p-STAT3, P53, and GPX4. (c) P53 mRNA levels was detected by qRT-PCR. (d–g) Overexpression of JAK2 reverses the changes in cell death, GSH, MDA, and cellular iron caused by AICAR and erastin. Independent experiments were repeated three times, and representative data were shown. Data shown represent mean ± SD from at least three independent experiments. Comparisons were performed using Student's *t*-test and one-way ANOVA. Fer-1: ferrostatin-1; ns: not significant. ^∗∗∗^*p* < 0.001 and ^###^*p* < 0.001.

**Figure 8:**
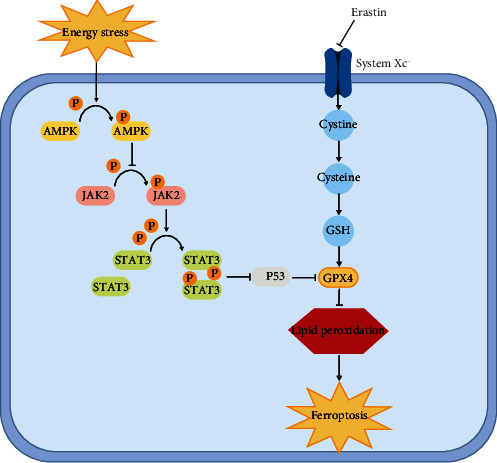
Schematic diagram displays potential mechanism of energy-stress-mediated promotion of erastin-induced ferroptosis.

STAT3 has been previously reported to negatively regulate ferroptosis [21], and the activation of the JAK2/STAT3 signaling pathway has been reported to inhibit P53 expression [22]. Thus, we explored the regulatory effect of the JAK2/STAT3 signaling pathway toward P53, which is involved in AMPK-mediated GPX4-dependent ferroptosis. We observed that after AMPK activation (in the glucose deprivation and AICAR groups), the phosphorylation levels of both JAK2 and STAT3 decreased significantly, whereas, after the overexpression of JAK2, the STAT3 phosphorylation level was restored, P53 expression reduced, and GPX4 expression increased significantly (Figures 7(a) and 7(b)), suggesting that AMPK activation regulates P53 expression via the JAK2/STAT3 signaling pathway. Moreover, the changes in the P53 mRNA levels suggest that the regulation of P53 by JAK2/STAT3 signaling occurred at the transcriptional level (Figure 7(c)). Findings from further experiments revealed that after the overexpression of JAK2, the enhancement of ferroptosis, reduction in GSH level, elevation in MDA level, and rise in cellular iron level in response to AMPK activation were ameliorated significantly (Figures 7(d)–7(g)). These results indicated that the regulation of the JAK2/STAT3 signaling pathway toward P53 contributes to AMPK-mediated GPX4-dependent ferroptosis (Figure 8).

## 4. Discussion

Glucose is a critical nutrient that is necessary for tumor cells to maintain normal metabolism and redox homeostasis. However, with rapid tumor growth, glucose deficiency-induced energy stress is a constant predicament for tumor cells. Glucose deficiency leads to redox system imbalance and energy metabolism deficiency in tumor cells, which further inhibits cell survival [23]. Ferroptosis is also primarily caused by lipid peroxidation owing to the dysregulation of the redox system in cells [5]. Therefore, we deduced that energy stress may promote ferroptosis in tumor cells. In the present study, we found that the simulation of energy stress with glucose deprivation increased erastin-induced cell death, besides decreasing the intracellular GSH level and increasing the MDA level. Combined with findings from the TEM images, energy stress was found to promote ferroptosis in renal cancer cells. Next, we found that AMPK phosphorylation activation increased after energy stress, whereas the expression of GPX4, the core enzyme in the regulation of ferroptosis, decreased. After GPX4 overexpression was induced, the level of ferroptosis induced by erastin and AMPK activation decreased significantly, indicating that energy stress-induced ferroptosis is associated with an AMPK activation-mediated decrease in GPX4 expression. Mechanistically, we observed that energy stress-induced AMPK activation inhibits GPX4 expression at the transcriptional level via P53 through the suppression of the JAK2/STAT3 signaling pathway, which consequently promotes ferroptosis.

Owing to the rapid proliferation of tumor cells, their energy metabolism undergoes adaptive shifts, such as the Warburg effect and pentose phosphate pathway (PPP) upregulation, to fulfill the energy demands for survival and biosynthesis [24, 25]. Thus, tumor-specific energy metabolism is the central force regulating redox homeostasis in tumor cells and a key factor in the regulation of ferroptosis [5]. Excessive growth inevitably leads to a relative deficiency of energy supply in the tumor microenvironment [26]. This forces tumor cells into a condition of energy stress, which leads to the inhibition of metabolic pathways, such as glycolysis and PPP, and thus affects tumor cell survival [27]. As the most important energy sensor, AMPK has largely been the focus of existing studies on energy stress and ferroptosis. However, the role of AMPK in the regulation of ferroptosis is controversial, considering evidence from recent studies. Lee et al. reported that AMPK activation during energy stress could block ferroptosis induced by cystine deficiency and GPX4 inhibition [28]. Mechanistically, energy stress activates AMPK, leading to the inhibition of downstream ACC by phosphorylation. Acetyl-CoA carboxylase (ACC) is a rate-limiting enzyme for fatty acid biosynthesis, which increases fatty acid synthesis and inhibits fatty acid oxidation. The inhibition of ACC activity decreases the intracellular PUFA levels and thereby reduces cellular susceptibility to ferroptosis. In addition to the effect on lipid synthesis, the inhibition of ACC caused by AMPK activation also reduces NADPH consumption and enhances resistance to ferroptosis [28]. LKB1, a serine/threonine kinase, is also involved in the regulation of metabolism during energy stress in tumor cells [29]. Li et al. found that LKB1 enhanced downstream AMPK and ACC phosphorylation and inhibited PUFA production, thereby limiting the cellular susceptibility to ferroptosis [30]. These findings suggest that the activation of AMPK during energy stress can effectively enhance cellular resistance to ferroptosis and enable tumor cells to survive in an adverse environment. However, contrary to the findings, Song et al. showed that AMPK-mediated BECN1 phosphorylation promotes ferroptosis by directly binding to SLC7A11 to form a complex inhibiting the activity of system Xc- [31]. Consistently, our results also showed that energy stress-mediated AMPK activation transcriptionally inhibited GPX4 to promote ferroptosis through the JAK2/STAT3/P53 axis. Taken together, we propose that the role of energy stress and AMPK on ferroptosis in cancer may be dependent on the context. Energy stress-mediated AMPK activation could either resist ferroptosis and promote tumor survival through the inhibition of unsaturated fatty acid synthesis or enhance ferroptosis and suppress tumor survival through the inhibition of protein biosynthesis or regulation of ferroptosis-related enzymes.

The JAK2/STAT3 signaling pathway is widely known to be involved in various biological processes, such as immunity, differentiation, cell death, and tumorigenesis [32]. In recent years, AMPK has been shown to inhibit the JAK/STAT signaling pathway through various mechanisms, participating in the regulation of inflammatory responses and tumor metabolism. He et al. found that the activation of AMPK inhibited STAT1-dependent inflammatory responses that protected against vascular inflammation and atherosclerosis [33]. In addition, the findings of this study suggested that AMPK inhibited STAT1 activation by inducing mitogen-activated protein kinase phosphatase-1. Nerstedt et al. successively confirmed that AMPK activation could inhibit liver inflammation and insulin resistance by suppressing the JAK/STAT signaling pathway [34, 35]. Besides, the inhibition of the mTOR pathway by AMPK was also reported to potentially contribute to the inhibition of the JAK/STAT signaling pathway [36]. In oncology research, Rutherford et al. identified that AMPK activation inhibited acute lymphoblastic leukemia by blocking the JAK/STAT signaling pathway through the inhibition of WT or JAK1^V658F^ [37]. Meanwhile, blockade of the JAK/STAT signaling pathway was also effective in limiting the inflammation-related side effects associated with current treatments. Studies on myeloproliferative neoplasms showed that AMPK activation could inhibit a series of JAK mutants, including JAK2^V617F^, and could thus effectively control antiproliferative effects and hemostatic dysfunction [38]. As evident, most current studies on the regulation of the JAK/STAT3 signaling pathway by AMPK focus on hematological tumors, probably because JAK mutations are relatively more prevalent in hematological tumors. Meanwhile, fewer studies have been conducted on solid tumors. In our study, AMPK activation could inhibit the phosphorylation of the JAK2/STAT3 signaling pathway and its functions in transcriptional regulation, which helped promote ferroptosis; however, the precise mechanism remains to be investigated further.

A growing body of evidence suggests that ferroptosis is a potential therapeutic target for tumor progression inhibition [20, 39, 40]. However, even though it has been established that tumor cells are often exposed to energy stress, the effect of energy stress on ferroptosis in renal cancer is yet to be reported. In the present study, we showed that energy stress-mediated AMPK activation could effectively promote ferroptosis in renal cancer via the JAK2/STAT3/P53 axis. We anticipate that the pharmacological activation of AMPK may exert a synergistic effect on chemotherapy with ferroptosis-inducing effects, facilitating a curative effect or reducing chemoresistance. Future studies will further investigate the effects of the interaction between AMPK and ferroptosis in tumor development.

## Data Availability

The datasets used and analyzed during the current study are available from the corresponding author on reasonable request.

## Supplementary Materials

Supplementary 1 Supplementary Figure 1: the effect of AMPK activation on apoptosis.

Supplementary 2 Supplementary Figure 2: another siAMPK was used to exclude off-target effect.

Supplementary 3 Supplementary Table 1: clinical characteristics of the TCGA-KIRC patients used in the study.
