# Supplementary figures and images for: Energy-Stress-Mediated AMPK Activation Promotes GPX4-Dependent Ferroptosis through the JAK2/STAT3/P53 Axis in Renal Cancer
Source: Oxid Med Cell Longev. 2022 Oct 4;2022:2353115. doi: 10.1155/2022/2353115 (PMC9554664; doi:10.1155/2022/2353115)

**A**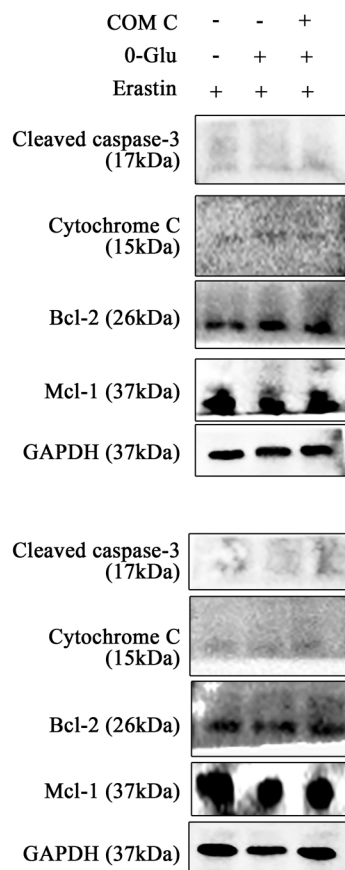**B**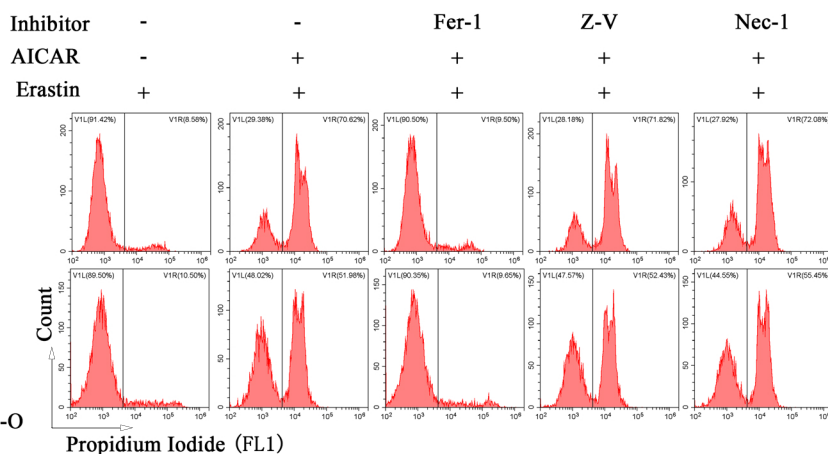**C**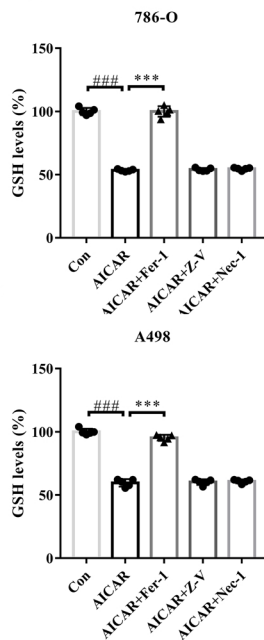**D**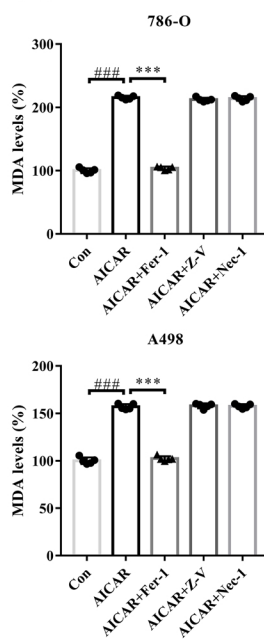**E**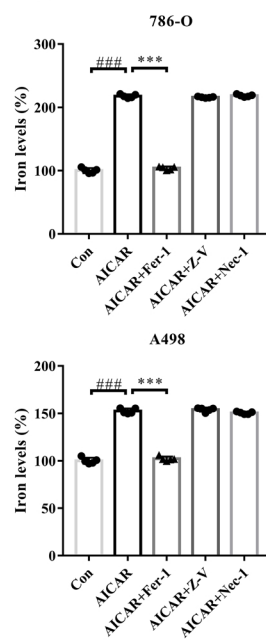**F**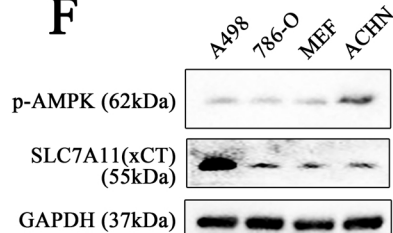**G**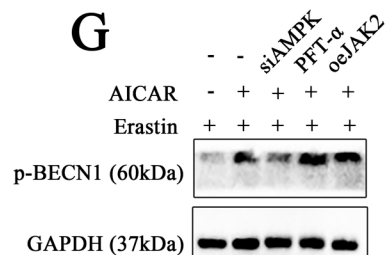

Supplement: Supplementary 1 — Supplementary Figure 1: the effect of AMPK activation on apoptosis. [file 2353115.f1.pdf]

# A

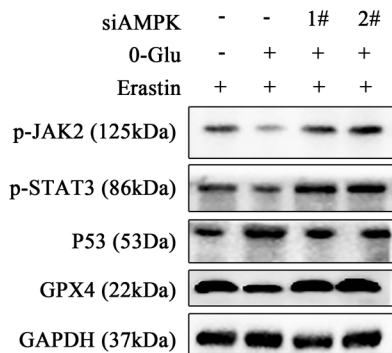

# B

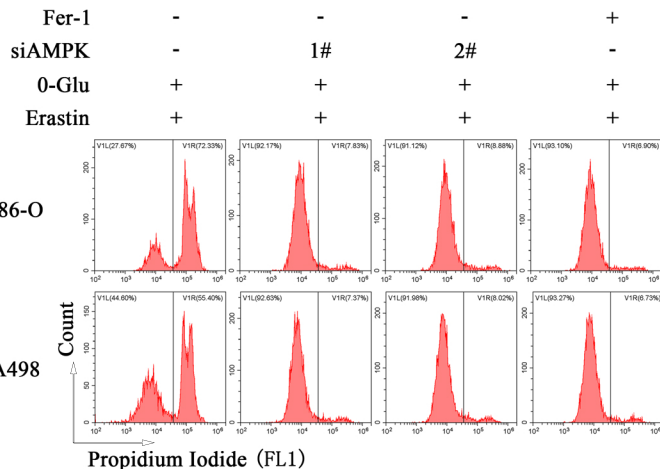

# C

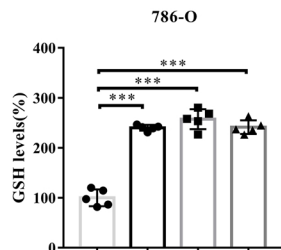

A498

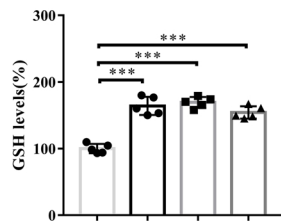

# D

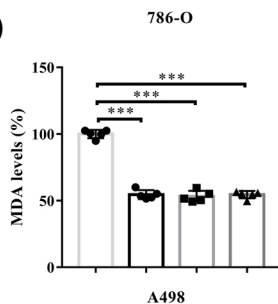

A498

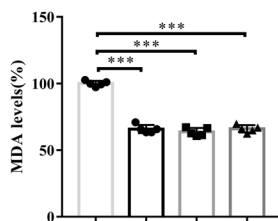

# E

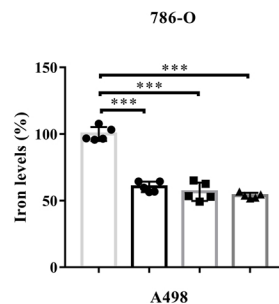

A498

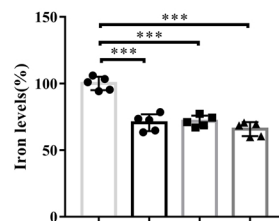

|         | Fer-1 | -  | -  | - | + |
|---------|-------|----|----|---|---|
| siAMPK  | -     | 1# | 2# | - | - |
| 0-Glu   | +     | +  | +  | + | + |
| Erastin | +     | +  | +  | + | + |

|         | Fer-1 | -  | -  | - | + |
|---------|-------|----|----|---|---|
| siAMPK  | -     | 1# | 2# | - | - |
| 0-Glu   | +     | +  | +  | + | + |
| Erastin | +     | +  | +  | + | + |

|         | Fer-1 | -  | -  | - | + |
|---------|-------|----|----|---|---|
| siAMPK  | -     | 1# | 2# | - | - |
| 0-Glu   | +     | +  | +  | + | + |
| Erastin | +     | +  | +  | + | + |

Supplement: Supplementary 2 — Supplementary Figure 2: another siAMPK was used to exclude off-target effect. [file 2353115.f2.pdf]
