# Supplementary material for: Energy-Stress-Mediated AMPK Activation Promotes GPX4-Dependent Ferroptosis through the JAK2/STAT3/P53 Axis in Renal Cancer
Source: Oxid Med Cell Longev. 2022 Oct 4;2022:2353115. doi: 10.1155/2022/2353115 (PMC9554664; doi:10.1155/2022/2353115)
Supplement: Supplementary 3 — Supplementary Table 1: clinical characteristics of the TCGA-KIRC patients used in the study. [file 2353115.f3.docx]

**Supplementary Table 1.** Clinical characteristics of the TCGA-KIRC patients used in the study.

|  | n |
| --- | --- |
| Gender |  |
| Male | 374 |
| Female | 185 |
| Age |  |
| ≤60 | 276 |
| >60 | 283 |
| neoplasm_histologic_grade |  |
| G1 | 11 |
| G2 | 239 |
| G3 | 223 |
| G4 | 86 |
| pathologic_M |  |
| M0 | 463 |
| M1 | 96 |
| pathologic_T |  |
| T1 | 268 |
| T2 | 76 |
| T3 | 202 |
| T4 | 13 |
| pathologic_stage |  |
| Stage I | 260 |
| Stage II | 62 |
| Stage III | 136 |
| Stage IV | 101 |
